# Variations in the Gut Bacteriome and Virome Associated with Cognitive Function in Puerto Rican Adults

**DOI:** 10.1002/alz70860_098660

**Published:** 2025-12-23

**Authors:** Deepika Dinesh, Xochitl Morgan, Tammy M Scott, Mahdi Garelnabi, Kelsey M Mangano, Sabrina E Noel, Curtis Huttenhower, Katherine L Tucker, Natalia Palacios

**Affiliations:** ^1^ Department of Public Health, University of Massachusetts Lowell, Lowell, MA, USA; ^2^ Harvard T.H. Chan School of Public Health, Boston, MA, USA; ^3^ Tufts University, Boston, MA, USA; ^4^ Department of Biomedical and Nutritional Sciences, University of Massachusetts Lowell, Lowell, MA, USA; ^5^ Harvard T. H. Chan School of Public Health, Boston, MA, USA

## Abstract

**Background:**

Gut bacterial variations and dysbiosis may influence cognitive function via the microbiome‐gut‐brain‐axis. Gut viruses may also, directly or indirectly, impact cognitive function by modulating the gut bacteria. Hispanics/Latinos, who may have unique microbiome characteristics, are at a higher risk of Alzheimer's disease and related dementia. There is a lack of research on the gut microbiome and, especially, the virome in Hispanics/Latinos. Here, we examined variations in the gut bacteriome and virome associated with cognitive function in the Boston Puerto Rican Health Study (BPRHS), a prospective cohort of older Puerto Rican adults residing in the Boston area.

**Method:**

This study was conducted in 316 BPRHS participants with fecal metagenomic sequencing and cognitive assessments, summarized as a composite global cognitive score (GCS). Taxonomic profiling of the gut bacteriome was performed using MetaPhlAN 4.0. Gut virome profiles from shotgun sequencing were generated using BAQLaVa 1.0. Cross‐sectional associations between bacterial and viral composition and GCS were assessed using alpha (Shannon) and beta (Bray‐Curtis) diversity indices. Feature‐wise testing was performed using multivariate linear regression (MaAsLin2) to identify bacterial and viral taxa associated with the GCS.

**Result:**

Among 316 participants (mean age 68.7 years, 70.9% female), there were no differences in overall bacterial or viral composition, measured by alpha and beta diversity, based on GCS. In feature‐wise analyses, adjusted for age, sex, and BMI, among participants with higher GCS (better cognitive function), we observed an enrichment of *Faecalibacterium prausnitzii* bacterium (β = 0.78, *p* = 0.01, FDR *p* = 0.22), and depletion of the phage *Carjivirus communis* (β = ‐1.07, *p* < 0.01, FDR *p* = 0.09).

**Conclusion:**

The observed results suggest an enrichment of *F. prausnitzii*, a beneficial butyrate producing taxa, among participants with better cognitive function, and enrichment of *Carjivirus communis*, a *Crassvirales* dsDNA *Bacteroidetes* phage, among participants with worse cognitive function. A recent study reported an association between *Bacteroidetes* phages and amyloid β and Alzheimer's disease pathology. Gut viral variations may modulate gut bacteria, impacting cognitive function. Future work will test interactions of the gut bacteriome, virome and their functional pathways, as related to cognitive function in Puerto Rican adults.

